# PXF-1 promotes synapse development at the neuromuscular junction in *Caenorhabditis elegans*

**DOI:** 10.3389/fnmol.2022.945680

**Published:** 2022-10-13

**Authors:** Reagan Lamb, Bithika Dhar, Salvatore J. Cherra

**Affiliations:** Department of Neuroscience, University of Kentucky College of Medicine, Lexington, KY, United States

**Keywords:** RapGEF, synaptic vesicles, GTPase, locomotor circuit, motor neuron, actin

## Abstract

Guanine nucleotide exchange factors (GEFs) are a family of proteins that modulate small G protein signaling. Mutations in a subfamily of GEFs that act on Rap, known as RapGEFs, have been associated with neurological disorders, and knockout mice display impairments in neuronal activity. However, the precise functions of RapGEFs in the nervous system remain unclear. Here, we have used the *Caenorhabditis elegans* neuromuscular junction, to investigate how the RapGEF homolog, PXF-1, regulates synaptic function. We found that loss of function mutations in *pxf-1* reduced cholinergic activity at the neuromuscular junction. We observed that PXF-1 is expressed in the nervous system, and its expression in neurons is sufficient to promote synaptic activity. In *pxf-1* mutant animals, there is a reduction in the levels of synaptic vesicles in cholinergic motor neurons but no change in the overall synapse numbers. In addition to synaptic vesicles proteins, we also found that filamentous actin, a scaffold for nascent synapses, was reduced at developing cholinergic synapses in *pxf-1* mutant animals. Our studies indicate that PXF-1 regulates neuromuscular function by promoting the formation of actin filaments to support the development of motor neuron synapses.

## Introduction

Nervous system function requires precise organization of presynaptic and postsynaptic terminals. Presynaptic protein complexes establish active zones at nerve terminals (Zhen and Jin, 1999; Kaufmann et al., 2002; Dai et al., 2006; Held et al., 2016; Radulovic et al., 2020). These presynaptic organizing complexes recruit synaptic vesicles and calcium channels to the active zones through a network of protein-protein interactions (Nonet et al., 1997; Biederer and Sudhof, 2000; Olsen et al., 2005; Mahoney et al., 2006; Weimer et al., 2006; Kushibiki et al., 2019; Zarebidaki et al., 2020; Oh et al., 2021). Additionally, SNAREs and associated proteins coordinate nerve terminal organization through the docking of synaptic vesicles at active zones (Deak et al., 2009; Vardar et al., 2016; Lai et al., 2017). The actin cytoskeleton also plays a role early in the establishment of active zones and during the recruitment or clustering of synaptic vesicles to presynaptic terminals (Zhang and Benson, 2001; Sun and Bamji, 2011; Stavoe and Colon-Ramos, 2012; Chia et al., 2014).

In addition to these presynaptic organizing complexes, signal transduction pathways coordinate the organization and development of presynaptic terminals. For example, GTPases, like Rap, Rac, or Rab regulate synaptic function, presynaptic organization, or synaptic vesicle cycling (Nonet et al., 1997; Morozov et al., 2003; Graf et al., 2009; Sun and Bamji, 2011; Stavoe and Colon-Ramos, 2012; Subramanian et al., 2013). The activity of GTPases is tightly regulated by guanine nucleotide exchange factors (GEFs). RapGEFs, like Epac proteins, are stimulated by cAMP and regulate the size of synaptic vesicle pools, neurotransmitter release, and synaptic plasticity (Kaneko and Takahashi, 2004; Gekel and Neher, 2008; Martin et al., 2020). In addition to the Epac subfamily, there is another subfamily of RapGEFs that are associated with neurological disorders (Bacchelli et al., 2003; Xu et al., 2008; Heo et al., 2018). In mice, deletions of RapGEF2 disrupt brain development (Bilasy et al., 2009, 2011; Maeta et al., 2016), and RapGEF6 knockout mice display neuronal activity impairments (Levy et al., 2015). Yet, the roles of non-Epac RapGEFs in regulating synaptic function have not been investigated.

Here, we used the *Caenorhabditis elegans* (*C. elegans*) motor circuit to investigate the roles of the PDZ guanine nucleotide exchange factor (PXF-1), a homolog of RapGEF2 and RapGEF6. Previous work indicated that RNAi knockdown of *pxf-1* reduced neuromuscular activity (Sieburth et al., 2005). Here, we found that PXF-1 functions in neurons to promote neuromuscular function. Loss of function mutations impaired synaptic vesicle clustering and reduced the levels of presynaptic filamentous actin (F-actin). These results show that PXF-1 regulates an actin-dependent mechanism that maintains synaptic vesicles at presynaptic terminals.

## Materials and methods

### Strains and transgenesis

All strains were maintained at 20°C on nematode growth medium (NGM) plates seeded with OP50 as previously described (Brenner, 1974). All information for strains used in this study is listed in Table 1. Transgenic animals harboring extrachromosomal arrays were generated using standard microinjection method (Mello et al., 1991). We used *Pmyo-2:mCherry* (2 ng/μl) or *Punc-122:RFP* (60 ng/μl) as coinjection markers. CRISPR/Cas9 gene editing was used to insert a 3xFLAG sequence into the C-terminus of the *pxf-1* genomic locus using a 3xFLAG-containing single-stranded oligonucleotide as previously described (Dokshin et al., 2018). All mutant or transgenic strains generated in this study were genotyped using PCR to detect deletion mutants or Sanger sequencing to confirm point mutations. Mutant alleles of *pxf-1* were backcrossed with N2 to generate 1X backcrossed alleles. Subsequently, 1X backcrossed animals were crossed with N2 to isolate 2X backcrossed mutant alleles of *pxf-1.* All subsequent crosses with fluorescent markers were initiated with the 2X backcrossed *pxf-1* mutant strain.

**TABLE 1:** Details and genotypes for the strains used in this study.

Strain	Genotype	Notes
N2	*wild type*	CGC257 from *Caenorhabditis* genetics center
SJC69	*pxf-1(gk955083) IV*	2X backcrossed from VC20736 with N2 (Thompson et al., 2013)
SJC77	*pxf-1(gk770584) IV*	2X backcrossed from VC40706 with N2 (Thompson et al., 2013)
SJC15	*Pmyo-3:GCaMP2(zwIs132)*	Allele made by Dr. Zhao-Wen Wang in ZW495 (Liu et al., 2011)
SJC315	*pxf-1(gk955083) IV; Pmyo-3:GCaMP2(zwIs132)*	
SJC283	*pxf-1:3xFLAG(blu32) IV*	
SJC349	*pxf-1(gk955083) IV; Prgef-1:pxf-1 cDNA(bluEx53)*	
SJC350	*pxf-1(gk955083) IV; Prgef-1:pxf-1 cDNA(bluEx54)*	
SJC376	*pxf-1(gk955083) IV; Pmyo-3:pxf-1 cDNA(bluEx68)*	
SJC377	*pxf-1(gk955083) IV; Pmyo-3:pxf-1 cDNA(bluEx69)*	
SJC182	*Punc-129:mCherry:RAB-3(tauIs46)*	Allele made by Dr. Jeremy Dittman in JSD97 and previously used (Zhou et al., 2017)
SJC324	*pxf-1(gk955083) IV; Punc-129:mCherry:RAB-3(tauIs46)*	
SJC394	*Punc-4:SNB-1:GFP(jsIs42) X*	Allele made by Dr. Michael Nonet in NM0670 (Nonet, 1999)
SJC395	*pxf-1(gk955083) IV; Punc-4:SNB-1:GFP(jsIs42) X*	
SJC10	*Punc-25:SNB-1:GFP(juIs1) IV*	Allele made by Dr. Yishi Jin in CZ333 (Zhen and Jin, 1999)
SJC397	*pxf-1(gk955083) IV; Punc-25:SNB-1:GFP(juIs1) IV*	
SJC39	*Punc-129:UNC-10:GFP(nuIs165) II*	Allele made by Dr. Joshua Kaplan in KP3928 (McEwen and Kaplan, 2008)
SJC223	*Punc-129:UNC-10:GFP(nuIs165) II; pxf-1(gk955083) IV*	
SJC316	*Punc-129:mCherry:RAB-3(tauIs46); Punc-17b:GFP:utrophin-CH(bluEx30)*	
SJC317	*pxf-1(gk955083) IV; Punc-129:mCherry:RAB-3(tauIs46); Punc-17b:GFP:utrophin-CH(bluEx30)*	
SJC450	*Punc-129:mCherry:RAB-3(tauIs46); Prgef-1:wve-1 (bluEx88)*	
SJC429	*pxf-1(gk955083) IV; Punc-129:mCherry:RAB-3(tauIs46); Prgef-1:wve-1 (bluEx88)*	
SJC430	*pxf-1(gk955083) IV; Punc-129:mCherry:RAB-3(tauIs46); Prgef-1:wve-1 (bluEx89)*	

### Behavioral assays

Plates containing aldicarb (1 mM, Sigma-Aldrich) or levamisole (0.5 mM, Sigma-Aldrich) were poured in batches and stored at 4°C until used. All genotypes were run in parallel for each experiment using plates poured in the same batch. L4 animals were moved to fresh NGM plates and incubated overnight. As day-one adults, animals were moved to plates containing 1 mM aldicarb or 0.5 mM levamisole. Animals were then touched every 15 min for 2 h on aldicarb or 90 min on levamisole to determine paralysis. Animals that did not respond after three touches were considered paralyzed. All assays were performed at room temperature (20–21°C), and individuals performing the assays were blinded to the genotypes prior to plating on aldicarb or levamisole.

### Fluorescence microscopy

All images were taken at 60X magnification using Ti-2E widefield fluorescence microscope equipped with a DS-Qi2 camera (Nikon). To image presynaptic terminals, we used mCherry:RAB-3, SNB-1:GFP, UNC-10:GFP, or endogenous UNC-17. To visualize actin filaments, we used mGFP fused to the calponin homology domain (CH) of the actin-binding protein utrophin (mGFP:ut-CH) (Burkel et al., 2007; Chia et al., 2012; Chen et al., 2018). Animals were immobilized in M9 buffer using a 10% agarose pad. With the exception of animals harboring *jsIs42*, images of the posterior synapses of the dorsal cord in L4 animals were captured using identical settings for each fluorescent protein. For *jsIs42*, we captured images of ventral synapses in SAB neurons. All image analysis was performed using the FIJI distribution of ImageJ software (Schindelin et al., 2012). The intensities of fluorescent markers were measured using a custom, semi-automated line-scan analysis script, in which a 10-pixel line was drawn to connect synaptic puncta along ∼30–40μm of dorsal cord. The intensity profile of the line was plotted, and the line tool was used to connect the valley points between each peak to calculate the area under the curve for each peak using the wand tool. The density of synaptic puncta was calculated as the number of peaks from the intensity profile divided by the measured length of dorsal cord. To calculate the intensity of presynaptic F-actin, we performed a line scan for mCherry:RAB-3 and mGFP:ut-CH channels and quantified the intensity of mGFP:ut-CH fluorescence within 1 μm of mCherry:RAB-3 peaks using a semi-automated custom script in ImageJ.

For imaging of the GCaMP marker, intact animals were immobilized on a 5% agarose pad with M9 and 100 nm polystyrene beads (Polybead Microspheres, Polysciences) (Kim et al., 2013). Images of the dorsal muscles were captured at 10 Hz for 5 min in the region contralateral to the vulva. To analyze GCaMP signal intensity, we created a uniform region of interest using ImageJ to measure the mean fluorescence intensity in the muscle. Fluorescence intensity was converted to ΔF/F and smoothed using a custom MATLAB script. Calcium transient peaks (ΔF/F ≥ 0.05) were counted and the area under each peak quantified using ImageJ. All analytical scripts and macros are available on GitHub in CherraLab repositories.

### DNA cloning and plasmid construction

PXF-1a cDNA (pREA4) was constructed using Gibson assembly (Gibson et al., 2009) of 3 gBlock Gene Fragments (Integrated DNA Technologies) containing overlapping segments and pSJC213, a modified pCR8-GW plasmid. WVE-1 cDNA (pBD11) was constructed using 1 gBlock Gene Fragment and pSJC213. For tissue-specific expression of *pxf-1a* cDNA, LR reactions were performed according to manufacturer’s specifications using Gateway LR Clonase II Enzyme Mix (Thermo Scientific) and destination vectors to produce pREA7 [P*rgef-1:pxf-1a cDNA*] and pREA17 [*Pmyo-3:pxf-1a cDNA*]. Pan-neuronal expression of WVE-1 cDNA was achieved using pBD16 [*Prgef-1:wve-1 cDNA*]. We also used LR Clonase II to move mGFP fused to the calponin homology domain of utrophin (ut-CH) into a destination vector containing the *unc-17b* promoter to create an F-actin marker pSJC235 [*Punc-17b:mGFP:ut-CH*].

### Whole-mount immunocytochemistry

To visualize endogenous localization of PXF-1, adult animals were washed in M9 buffer and then washed in distilled water prior to spreading worms on poly-L-lysine-coated slides as previously described (Duerr et al., 1999). A second poly-L-lysine-coated slide was used to sandwich the worms. The slide sandwich was immediately frozen using a heat block placed in a bath of liquid nitrogen and then placed at −80°C for 20 min. The slides were separated, and the samples fixed in ice cold methanol for 2 min, followed by ice cold acetone for 4 min. The slides were washed in PBS, and then incubated with 5% bovine serum albumin (BSA) in PBS with Triton X-100 (PBST) for 1 h at room temperature. Samples were incubated overnight with antibodies against DYKDDDDK (RRID:AB 1625981, 1:50, Novus Biologicals) and UNC-10 (1:50, Developmental Studies Hybridoma Bank) in 5% BSA in PBST. After washing with 2% BSA in PBST, samples were incubated for 1 h with fluorescent secondary antibodies: Alexa Fluor 488 donkey anti-rat and Alexa Fluor 594 donkey anti-mouse (Invitrogen). Slides were washed in 2% BSA in PBST and then in PBS. Coverslips were mounted onto the slides using mounting media (VectaShield).

To visualize endogenous UNC-17, we fixed and processed worms as previously described with minor modifications (Cherra and Jin, 2016). Samples were blocked with 5% BSA for 1 h and then incubated overnight with an UNC-17 antibody (1:500, Developmental Studies Hybridoma Bank). Samples were washed with 2% BSA in PBST and incubated for 1 h with Alexa Fluor 594 donkey anti-mouse secondary. After washing with 2% BSA in PBST and a PBS wash, samples were mixed with mounting media, spread on a microscope slide, and covered with a coverslip.

### Statistical analysis

All statistical analyses were performed using Prism 9 (Graphpad). For comparisons between two groups, we used a *t*-test where a *p*-value less than 0.05 was considered significant. We used a Mann-Whitney test for non-parametric data. For comparisons between multiple groups with normally distributed data, a one-way ANOVA was performed followed by Sidak’s multiple comparisons test. For data that was not normally distributed, a Kruskal-Wallis test was used followed by Dunn’s multiple comparisons test. We used a Mixed-effects analysis followed by Dunnett’s multiple comparisons for drug-induced paralysis timecourses. For multiple comparisons, a corrected *p*-value less than 0.05 was considered significant.

## Results

### Mutations in *pxf-1* reduce neuromuscular junction function

**FIGURE 1:**
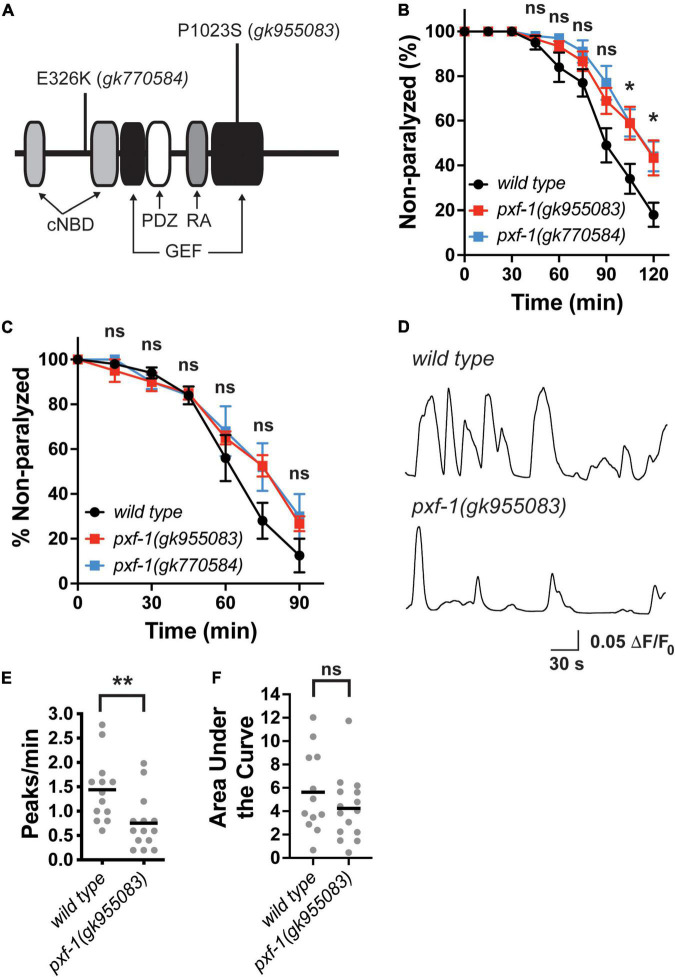
Aldicarb sensitivity and postsynaptic calcium transients are reduced in *pxf-1* mutant animals. **(A)** Diagram of predicted PXF-1 protein. The *gk955083* and *gk770584* alleles are indicated above the protein. The predicted functional domains and motifs are indicated below the protein. cNBD is a cyclic nucleotide-binding domain, GEF is a guanine nucleotide exchange factor domain, PDZ is PSD95/Disk Large/ZO-1 domain, and RA is a Ras interacting domain. **(B)** Quantification of non-paralyzed animals during 120 min on 1 mM aldicarb. Dots indicate means, and error bars indicate the SEM. *n* = 7–10 trials. *p < 0.05 or ns = not significant between either mutant and wild type animals at each timepoint. **(C)** Quantification of non-paralyzed animals during 90 min on 0.5 mM levamisole. Dots indicate means, and error bars indicate the SEM. *n* = 4–5 trials. ns = not significant at each timepoint. **(D)** Representative traces of GCaMP intensity in dorsal muscles in animals immobilized with polystyrene beads. Peaks greater than 0.05 ΔF/F_0_ were analyzed in panels **(E,F) (E)** Quantification of the number of peaks per minute. Gray dots indicate individual animals, and black bars indicate the means. *n* = 12–15 animals. **p < 0.01. **(F)** Quantification of the area under each peak. Gray dots indicate individual animals, and black bars indicate the means. *n* = 12–15 animals. ns, not significant.

Since RapGEFs have been associated with neurological disorders, we sought to determine how this family of proteins modulates nervous system function by focusing on a *C. elegans* RapGEF homolog, PXF-1. A previous RNAi screen indicated that knock-down of *pxf-1* caused animals to display resistance to aldicarb (Sieburth et al., 2005). However, previously identified deletions in *pxf-1*, which likely represent null mutations, displayed late larval or early adult lethality (Pellis-van Berkel et al., 2005). Therefore, we sought to identify viable putative loss-of-function mutations by screening previously generated missense mutations (Thompson et al., 2013). We performed two sequential backcrosses of *pxf-1* mutant-containing strains with wild type animals and identified two alleles of *pxf-1* that displayed resistance to aldicarb as compared to wild type animals (Figures 1A,B). The *gk770584* allele results in an E326K amino acid change. The *gk955083* allele results in a P1023S mutation in the guanine nucleotide exchange factor domain (Figure 1A). Since these mutations are viable as homozygotes, it suggests that these mutations likely represent weaker loss of function mutations as compared to null alleles.

To determine if this motor circuit defect detected as aldicarb resistance resulted from deficits in neuronal function or muscle function, we measured levamisole-induced paralysis (Figure 1C). Levamisole is a cholinergic receptor agonist for a subset of postsynaptic receptors expressed in the muscle (Brenner, 1974; Lewis et al., 1980). Incubation with levamisole leads to paralysis by directly overactivating the muscle cells (Richmond and Jorgensen, 1999). We observed no difference in the response to levamisole between either *pxf-1* mutant and wild type animals. For the remainder of our studies, we have focused on the *gk955083* allele that occurs in the GEF domain and likely reduces GEF function.

To determine if PXF-1 regulates basal neuromuscular junction (NMJ) function, we used a previously described genetically encoded calcium indicator, GCaMP2, to measure calcium transients in muscle cells (Liu et al., 2011). We immobilized intact animals with polystyrene beads and recorded GCaMP intensity in dorsal muscles. The *pxf-1(gk955083); zwIs132* mutant animals displayed a decrease in the frequency of calcium transients but no change in the area under the curve for each peak (Figures 1D–F). Together these data suggest that PXF-1 regulates neuronal function in motor neurons or upstream interneurons in the motor circuit.

### PXF-1 functions in neurons to promote neuromuscular junction function

**FIGURE 2:**
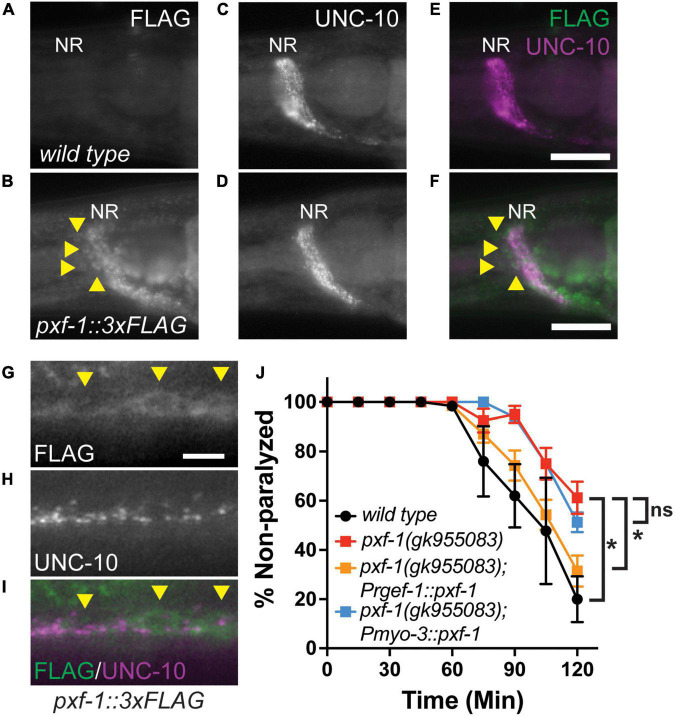
PXF-1 is expressed and functions in neurons. **(A,B)** FLAG staining, **(C,D)** UNC-10 staining, and **(E,F)** merged channels of heads of adult wild type and *pxf-1:3xFLAG* animals. NR indicates nerve ring. Arrowheads indicate neuron cell bodies. Scale bars are 25 μm. **(G)** FLAG staining, **(H)** UNC-10 staining, and **(I)** merged channels of ventral cord of *pxf-1:3xFLAG* adult animals. Arrowheads indicate motor neuron cell bodies. Scale bars are 5 μm. **(J)** Quantification of non-paralyzed animals during 120 min of exposure to 1 mM aldicarb. Transgenic PXF-1 cDNA was expressed using neuronal *rgef-1* and muscle *myo*-3 promoters. Dots represent means, and error bars indicate SEM. *n* = 6–8 trials. *p < 0.05; ns, not significant at 120 min.

To determine in which tissues PXF-1 may be acting for NMJ function, we generated C-terminal PXF-1:3xFLAG animals using CRISPR/Cas9-mediated genome engineering. We then stained for FLAG and the presynaptic active zone marker UNC-10 in wild type and *pxf-1:3xFLAG(blu32)* animals. PXF-1:3xFLAG was visible in the nerve ring labeled by UNC-10 (Figures 2A–F). In the ventral nerve cord, FLAG staining also was observed in axon/dendrites and the cell bodies of motor neurons (Figures 2G–I).

To determine where PXF-1 is required to promote neuromuscular function, we expressed *pxf-1* cDNA in neurons or body wall muscle using the *rgef-1* or *myo-3* promoters, respectively. Neuronal expression of *pxf-1* cDNA in *pxf-1(gk955083)* animals was sufficient to restore aldicarb sensitivity to wild type levels (Figure 2J). However, expression of *pxf-1* cDNA in muscle did not increase aldicarb sensitivity in *pxf-1(gk955083)* animals (Figure 2J), suggesting that PXF-1 function in neurons to promote NMJ function.

### *pxf-1* mutants reduce synaptic vesicle abundance

**FIGURE 3:**
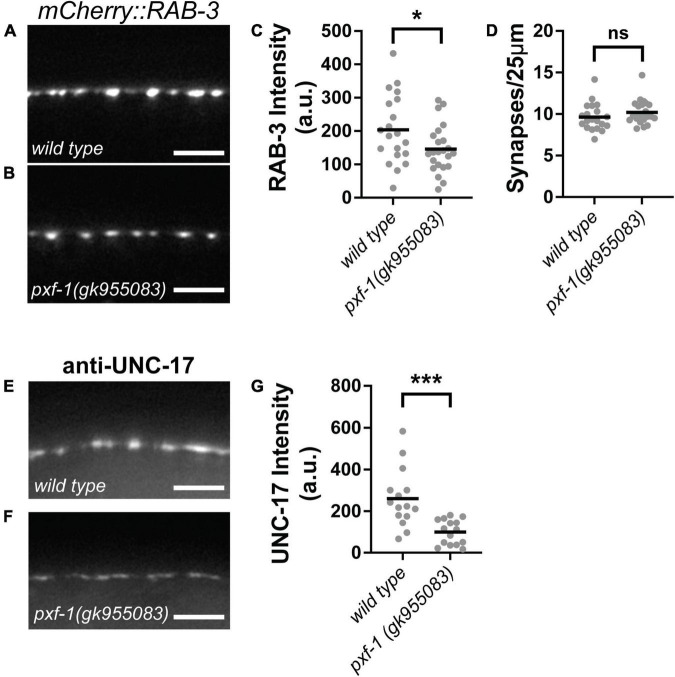
PXF-1 increases synaptic vesicle abundance in cholinergic motor neurons. **(A,B)** Representative images of mCherry:RAB-3 labeled acetylcholine synapses in dorsal cord of **(A)** wild type and **(B)** *pxf-1(gk955083)* animals. Scale bars are 5 μm. **(C)** Quantification of fluorescence intensity in arbitrary units, a.u. **(D)** Quantification of synaptic density. Gray dots represent individual animals, black bars indicate means. *n* = 20–23 animals. *p < 0.05; ns, not significant. **(E,F)** Representative images of dorsal cord stained for UNC-17 in **(E)** wild type and **(F)** *pxf-1(gk955083)* animals. Scale bars are 5 μm. **(G)** Quantification of fluorescence intensity. Gray dots represent individual animals, black bars indicate means. *n* = 15–16 animals. ***p < 0.001.

Since PXF-1 is required in neurons, we sought to determine whether *pxf-1* mutants displayed any defects in the number of synapses or abundance of synaptic markers. We used the synaptic vesicle marker, mCherry:RAB-3, to assess these parameters in cholinergic motor neurons. We observed a reduction in mCherry:RAB-3 intensity in *pxf-1(gk955083)* mutants as compared to wild type animals (Figures 3A–C). However, there were no differences in the number of synapses labeled by cholinergic mCherry:RAB-3 between wild type and *pxf-1(gk955083)* animals (Figures 3A,B,D). To further confirm that *pxf-1* mutants have a reduction in cholinergic synaptic vesicles, we stained for the endogenous vesicular acetylcholine transporter, UNC-17, in wild type and *pxf-1* mutant animals. Similar to the transgenic synaptic vesicle marker, *pxf-1* mutants displayed a decrease in UNC-17 staining in the dorsal cord (Figures 3E–G).

**FIGURE 4:**
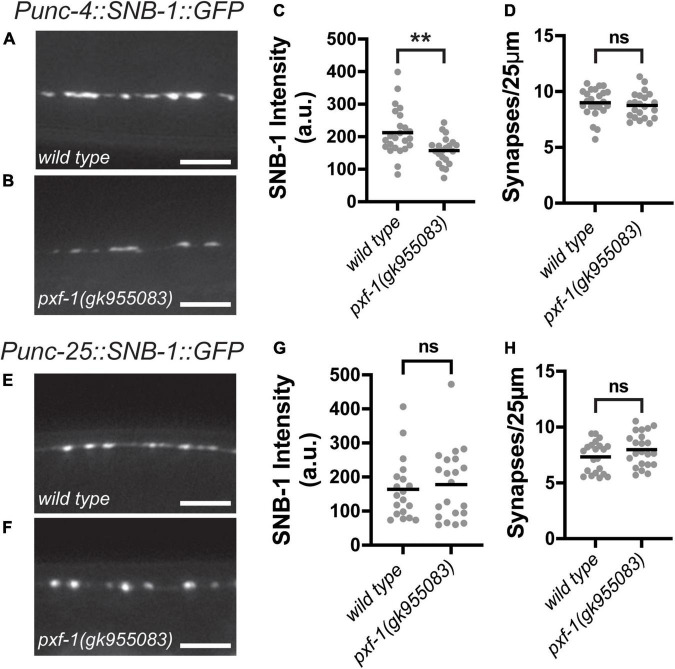
Synaptic vesicle abundance in GABAergic neurons is normal in *pxf-1* mutants. **(A,B)** Representative images of SNB-1:GFP labeled acetylcholine synapses in SAB neurons of **(A)** wild type and **(B)** *pxf-1(gk955083)* animals. Scale bars are 5 μm. **(C)** Quantification of fluorescence intensity in arbitrary units, a.u. **(D)** Quantification of synaptic density. Gray dots represent individual animals, black bars indicate means. *n* = 21–24 animals. **p < 0.01; ns, not significant. **(E,F)** Representative images of SNB-1:GFP labeled GABA synapses in dorsal cord of **(E)** wild type and **(F)** *pxf-1(gk955083)* animals. Scale bars are 5 μm. **(G)** Quantification of fluorescence intensity. **(H)** Quantification of synaptic density. Gray dots represent individual animals, black bars indicate means. *n* = 21–22 animals. ns, not significant.

Since GABAergic and cholinergic motor neurons synapse onto body wall muscles, we sought to determine if PXF-1 regulation of presynaptic terminals is a general mechanism to regulate synapse development or if PXF-1 mediates a neuron-specific mechanism to modulate cholinergic synapses. To analyze GABAergic and cholinergic synapses using the same synaptic vesicle marker, we compared wild type and *pxf-1(gk955083)* animals expressing SNB-1:GFP (*jsIs42*) in the cholinergic SAB neurons and SNB-1:GFP (*juIs1*) expressed in GABAergic motor neurons. Like mCherry:RAB-3, *pxf-1(gk955083)* animals displayed a reduction in SNB-1:GFP intensity in cholinergic motor neurons but no differences in synapse number (Figures 4A–D). In GABAergic motor neurons, we observed no differences in SNB-1:GFP intensity or synapse density between wild type and *pxf-1(gk955083)* animals (Figures 4E–H). Together, these data support the hypothesis that PXF-1 acts in a neuron-specific manner to promote synaptic development.

**FIGURE 5:**
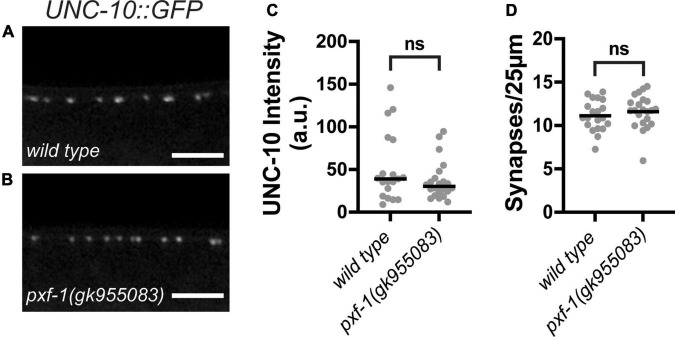
PXF-1 does not influence active zones. **(A,B)** Representative images of UNC-10:GFP labeled acetylcholine synapses in dorsal cord of **(A)** wild type and **(B)** *pxf-1(gk955083)* animals. Scale bars are 5 μm. **(C)** Quantification of fluorescence intensity in arbitrary units, a.u. Gray dots represent individual animals, black bars indicate medians. **(D)** Quantification of synaptic density. Gray dots represent individual animals, black bars indicate means. *n* = 19–22 animals. ns, not significant.

Active zone proteins cluster synaptic vesicles at presynaptic terminals. To determine if the reduction in synaptic vesicle markers caused by *pxf-1* mutations is due to deficiencies in active zone formation, we assessed the number cholinergic active zones and the intensity of UNC-10:GFP expressed in cholinergic motor neurons. UNC-10 is a RIM homolog that helps tether synaptic vesicles to the active zone (Weimer et al., 2006). Although *pxf-1(gk955083)* animals displayed reductions in synaptic vesicle markers, there were no differences in intensity or number of UNC-10:GFP puncta between wild type and *pxf-1(gk955083)* animals (Figures 5A–D). These data suggest that PXF-1 does not influence active zone assembly.

### *pxf-1* mutations reduce perisynaptic F-actin

**FIGURE 6:**
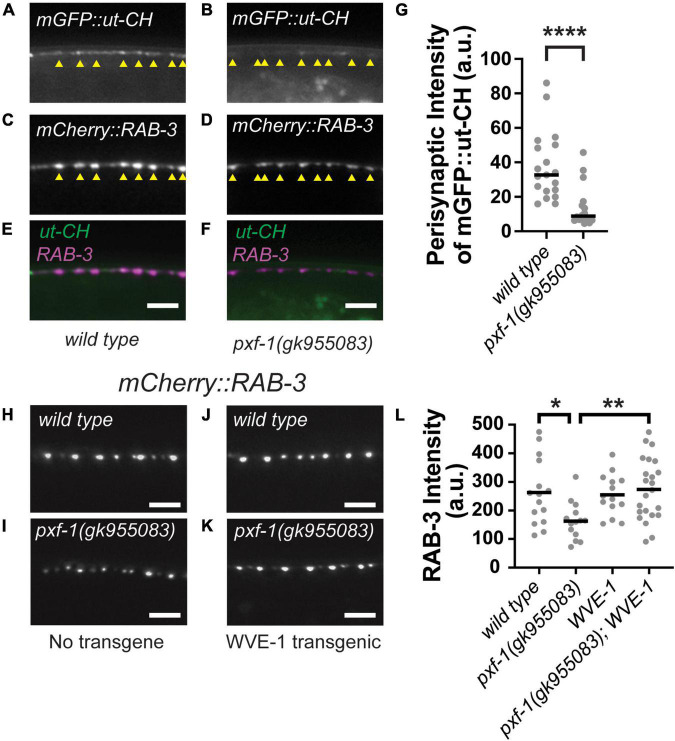
Perisynaptic mGFP:ut-CH is reduced in *pxf-1* mutant animals. **(A–F)** Representative images of **(A,B)** mGFP labeled calponin homology domain of F-actin binding protein utrophin, **(C,D)** mCherry:RAB-3 labeled acetylcholine synapses, and **(E,F)** merged channels in dorsal cord of wild type and *pxf-1(gk955083)* mutants. Yellow arrowheads indicate position of mCherry:RAB-3 labeled presynaptic terminals. Scale bars are 5 μm. **(G)** Comparison of intensities of mGFP:ut-CH puncta within 1 μm of mCherry:RAB-3 puncta measured in arbitrary units, a.u. Gray dots represent individual animals, black bars indicate medians. *n* = 16–19 animals. ****p < 0.0001. **(H–K)** Representative images of mCherry:RAB-3 labeled acetylcholine synapses in wild type and *pxf-1(gk955083)* animals in the presence or absence of *Prgef-1:wve-1* cDNA transgene. Scale bars are 5 μm. **(L)** Quantification of mCherry:RAB-3 from the indicated genotypes. Gray dots represent individual animals, black bars indicate means. *n* = 14–23. *p < 0.05; **p < 0.01.

In addition to active zone scaffolds, synaptic vesicle clustering is regulated by the actin cytoskeleton (Zhang and Benson, 2001; Sun and Bamji, 2011; Stavoe and Colon-Ramos, 2012). Since *pxf-1* mutants displayed no changes in active zone protein intensity, we hypothesized that PXF-1 may be acting through the actin cytoskeleton to promote synaptic vesicle abundance at nerve terminals. To visualize changes in F-actin, we created animals that express an mGFP labeled calponin homology domain of the F-actin binding protein utrophin (mGFP:ut-CH) and the RAB-3:mCherry synaptic marker (Figures 6A–F). We quantified the intensity of mGFP:ut-CH at presynaptic terminals labeled by RAB-3:mCherry and found that *pxf-1 (gk955083)* mutant animals showed a reduction in perisynaptic F-actin as compared to wild type animals (Figures 6A,B,G).

Multiple actin regulatory proteins coordinate the growth and stability of actin filaments, including WAVE and WASP, which act as scaffolds to promote actin polymerization (Pollard, 2007). In *C. elegans*, WAVE/wve-1 and WASP/wsp-1 cooperate to promote proper axon guidance in sensory neurons (Shakir et al., 2008). Loss of *wve-1* also disrupts perisynaptic pools of F-actin and reduces the abundance of presynaptic proteins (Chia et al., 2014). Since WVE-1 promotes F-actin levels, we reasoned that increasing WVE-1 levels in neurons could restore synaptic vesicle abundance in *pxf-1* mutants if the deficits in synaptic vesicle abundance were due to reductions in F-actin. To test this hypothesis, we expressed *wve-1* cDNA in wild type and *pxf-1* mutant neurons using the *rgef-1* promoter (Figures 6H–L). We observed that the intensity of cholinergic mCherry:RAB-3 was significantly increased in *pxf-1* mutants expressing *wve-1* cDNA as compared to *pxf-1* mutants expressing no transgene (Figures 6I,K). While overexpression of *wve-1* cDNA did not affect the intensity of mCherry:RAB-3 in wild type animals, it restored mCherry:RAB-3 intensity to wild type levels in the *pxf-1* mutants. Altogether these data suggest that PXF-1 promotes the formation or stability of perisynaptic actin filaments and that the synaptic deficit in *pxf-1* mutants can be ameliorated by overexpression of WVE-1, which is a known activator of actin filament formation.

## Discussion

Previous work had uncovered alterations in basal and fear-conditioned neural activation in RapGEF6 knockout animals (Levy et al., 2015). Deletion of RapGEF6 displayed both increases and decreases in neural activity in different brain regions (Levy et al., 2015), suggesting that RapGEF6 signaling mediates neuron-specific or circuit-specific events that underlie proper neuronal and circuit function. Studies in *Drosophila* suggest that the RapGEF6 homolog, Gef26, also participates in divergent downstream signaling pathways that mediate the growth or restraint of synapse size in the same neurons (Heo et al., 2017; Ou et al., 2019). Here we found that the *C. elegans* homolog, PXF-1, promotes cholinergic synapse development but has no effect on GABAergic synapse development. This suggests that homologs of RapGEF6 also participate in neuron-specific signaling pathways that govern circuit development and function. Further studies will be required to determine the mechanisms that mediate the neuron-specific signaling of RapGEF6 and its homologs and to understand whether these GEFs act through divergent signaling pathways to control neuron development as observed in *Drosophila*.

Mutations in *pxf-1* reduced synaptic vesicle markers without changes in active zone formation. During early phases of synaptic development, actin filaments are necessary to promote synaptic vesicle clustering (Zhang and Benson, 2001; Chia et al., 2014). Therefore, we hypothesize that PXF-1 signaling may regulate synaptic vesicle abundance independent of the formation of active zones. Based on our data, we would suggest a mechanism where PXF-1 promotes stability of the presynaptic actin cytoskeleton to retain synaptic vesicles at nerve terminals prior to docking with active zone scaffolds. Since loss of *pxf-1* reduces mGFP:ut-CH intensity, and possibly F-actin, in perisynaptic and intersynaptic regions, additional mechanisms may be involved. For example, perisynaptic actin filaments lateral to release sites are involved in the recycling of synaptic vesicles after exocytosis (Shupliakov et al., 2002; Bloom et al., 2003). Additionally, intersynaptic actin filaments may support axonal trafficking of recycled synaptic vesicles between neighboring synapses (Gramlich and Klyachko, 2017; Chenouard et al., 2020). Future studies will be required to decipher which of these mechanisms underlies the deficits in synaptic vesicle abundance observed in *pxf-1* mutants.

Although the current study supports a role of actin regulation downstream of PXF-1, the exact mechanism is not clear. Outside the nervous system, Rap GTPases, the direct targets of RapGEFs, stimulate actin polymerization (Taira et al., 2004; Lin et al., 2008; Mun and Jeon, 2012). However, other studies indicate that Rac signaling modulates actin filaments to promote the clustering of synaptic vesicles at presynaptic terminals (Sun and Bamji, 2011; Stavoe and Colon-Ramos, 2012). In the future, it will be important to determine precisely how PXF-1 signaling modulates the actin cytoskeleton, where this regulation occurs, and if RapGEF signaling influences the roles of Rap, Rac, or additional GTPases at the synapse.

Seminal studies have identified essential mechanisms that govern synapse formation and active zone organization. Yet, it’s not clear how these mechanisms are modulated during development, aging, or periods of plasticity. Small G proteins, like Rac, Rho, or Rap, promote the development of dendrites (Chen et al., 2005; Sfakianos et al., 2007; Cheng et al., 2021). Additionally, these small G proteins coordinate the placement and development of presynaptic terminals (Sun and Bamji, 2011; Stavoe and Colon-Ramos, 2012; Imai et al., 2016; Chen et al., 2018). Since small GTPases act as signaling hubs downstream of receptors and signal transduction pathways that coordinate growth and development, these proteins are well-positioned to coordinate synaptic connectivity and maturation during organismal development. However, the regulation of small G proteins and their signaling networks is not completely understood. Further studies into the intricacies of these pathways will provide a deeper understanding of the mechanisms that coordinate synapse development and neural circuit function.

## Data availability statement

The original contributions presented in this study are included in the article/supplementary material, further inquiries can be directed to the corresponding author.

## Author contributions

RL and SJC conceived and designed the experiments and wrote the manuscript. All authors gathered and analyzed the data and approved the submitted version.
